# Metabolic Reprogramming in Chloroplasts under Heat Stress in Plants

**DOI:** 10.3390/ijms19030849

**Published:** 2018-03-14

**Authors:** Qing-Long Wang, Juan-Hua Chen, Ning-Yu He, Fang-Qing Guo

**Affiliations:** 1 The National Key Laboratory of Plant Molecular Genetics, Institute of Plant Physiology & Ecology, Chinese Academy of Sciences, 300 Fenglin Road, Shanghai 200032, China; qlwang@sibs.ac.cn (Q.-L.W.); jhchen02@sibs.ac.cn (J.-H.C.); nyhe@sibs.ac.cn (N.-Y.H.); 2 CAS Center for Excellence in Molecular Plant Sciences, Institute of Plant Physiology & Ecology, Chinese Academy of Sciences, 300 Fenglin Road, Shanghai 200032, China

**Keywords:** heat stress, metabolic reprogramming, chloroplasts, chlorophyll breakdown, reactive oxygen species (ROS), antioxidant defense, protein turnover, Photosystem II (PSII) core subunit, PSII repair cycle, carbon assimilation

## Abstract

Increases in ambient temperatures have been a severe threat to crop production in many countries around the world under climate change. Chloroplasts serve as metabolic centers and play a key role in physiological adaptive processes to heat stress. In addition to expressing heat shock proteins that protect proteins from heat-induced damage, metabolic reprogramming occurs during adaptive physiological processes in chloroplasts. Heat stress leads to inhibition of plant photosynthetic activity by damaging key components functioning in a variety of metabolic processes, with concomitant reductions in biomass production and crop yield. In this review article, we will focus on events through extensive and transient metabolic reprogramming in response to heat stress, which included chlorophyll breakdown, generation of reactive oxygen species (ROS), antioxidant defense, protein turnover, and metabolic alterations with carbon assimilation. Such diverse metabolic reprogramming in chloroplasts is required for systemic acquired acclimation to heat stress in plants.

## 1. Introduction

Photosynthesis is thought to be the most important photo-chemical reaction, during which sunlight is trapped and converted into biological energy in plants. In general, leaves function as a highly specialized organ that are basically appointed in the photosynthetic process in higher plants. Leaf photosynthesis is substantially affected, often lethally, by high temperature stress, usually 10–15 °C above an optimum temperature for plant growth as plants are not capable of moving to more favorable environments [1,2,3]. Housed in chloroplasts, the photosynthetic apparatus is susceptible to be damaged by heat stress and the chloroplasts have been demonstrated to play an essential role in activation of cellular heat stress signaling [4,5,6]. Given that Photosystem II (PSII) is the most susceptible target within the chloroplast thylakoid membrane protein complexes for heat stress, heat stress commonly causes severe thermal damages to PSII, dramatically affecting photosynthetic electron transfer and ATP synthesis [1,2,3,7,8]. Under heat stress, heat stress-induced damages lead to alterations of photochemical reactions in thylakoid lamellae of chloroplast, reflected as a significant reduction in the ratio of variable fluorescence to maximum fluorescence (*Fv*/*Fm*) [1,2,9,10,11]. Exposure to high temperature stress causes oxidative stress in plants, particularly leading to the dissociation of oxygen evolving complex (OEC) in PSII, which further results in inhibition of the electron transportation from OEC to the acceptor side of PSII [1,2,12,13]. Heat stress causes cleavage of the reaction center-binding protein D1 of PSII in spinach thylakoids and induces dissociation of a manganese (Mn)-stabilizing 33-kDa proteins from PSII reaction center complex [14]. As a photosynthetic carbon fixation cycle, the Calvin–Benson cycle is responsible for the fixation of CO_2_ into carbohydrates, as well as assimilation, transport, and utilization of photoassimilates as the organic products of photosynthesis. In addition to the disruption of OEC in PSII, heat stress also results in dysfunction in the system of carbon assimilation metabolism in the stroma of chloroplast [2,8]. It has been observed that the disruption of electron transport and inactivation of the oxygen evolving enzymes of PSII dramatically inhibit the rate of ribulose-1,5-bisphosphate (RuBP) regeneration [10,15]. Heat stress-induced inhibition in the activity of ribulose-1,5-bisphosphate carboxylase/oxygenase (Rubisco) mainly results from inactivation of Rubisco activase that is extremely sensitive to heat stress because the enzyme Rubisco of higher plants is heat-stable [8,15]. In addition to the early effects on photochemical reactions and carbon assimilation, alterations in the microscopic ultrastructures of chloroplast and the integrity of thylakoid membranes were reported to be severely disrupted, including membrane destacking and reorganization when subjected to heat stress [2,16,17,18,19].

**Figure 1:**
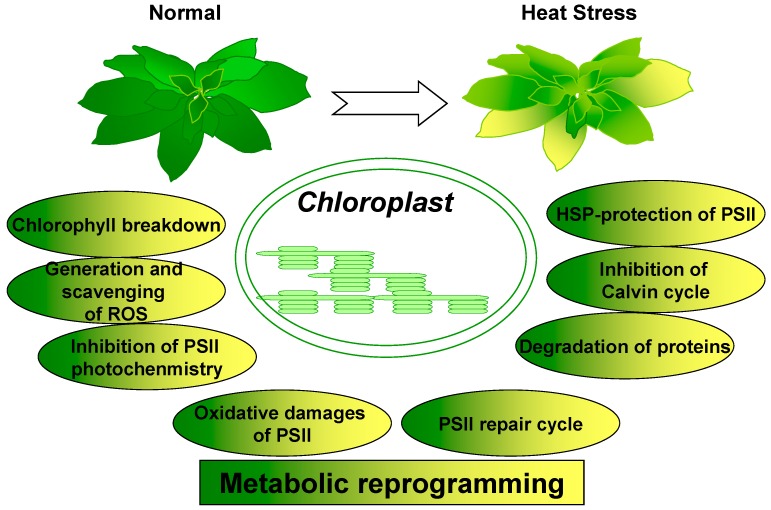
Extensive and transient metabolic reprogramming in chloroplasts under heat stress. Major events of metabolic reprogramming in response to heat stress include chlorophyll breakdown, generation of reactive oxygen species (ROS), antioxidant defense, protein turnover, and metabolic alterations with carbon assimilation. With respect to the systemic acquired acclimation to heat stress in plants, diverse metabolic reprogramming in chloroplasts is required for optimizing plant growth and development during high temperature stresses.

In photosynthetic organisms, chloroplasts respond to a variety of environmental stresses, including heat stress, with adjustments for major metabolic processes to optimize carbon fixation and growth requirements [2,3,6,8,20]. As one of the subcellular energy organelles, chloroplast in plant cells conducts major metabolic reprogramming processes including chlorophyll breakdown, generation and scavenging of reactive oxygen species (ROS), protein turnover, and metabolic alterations of carbon assimilation in response to heat stress (Figure 1). Here we review studies that have investigated the effects of heat stress on photosynthesis and its associated metabolic adaptations for optimizing plant growth and development under stress conditions. We will assess the role of chloroplast from an organellar perspective to begin building insights into better understanding the importance and significance of metabolic reprogramming within this organelle during high temperature stresses.

## 2. Chlorophyll Breakdown under Heat Stress

Chlorophyll (Chl) functions in harvesting light energy and driving electron transfer during the initial and indispensable processes of photosynthesis, and is a pigment consisting of two moieties: a chlorin ring containing a magnesium ion at its center and a long hydrophobic phytol chain, joined by an ester bond. It should be noted that when the photosynthetic apparatus is overexcited, and oxygen receives the absorbed energy from Chl, Chl acts as a harmful molecule, negatively affecting plant cells including most porphyrins [21,22]. Importantly, the breakdown of Chl is a physiological process as a prerequisite for protecting plant cells from hazardous effects of phototoxic pigments in association with recycling nitrogen sources from Chl-binding proteins in chloroplasts when leaves are senescing [22,23,24,25].

Recent studies have provided advances in better understanding of the pathway of Chl catabolism in higher plants. In brief, the removal of the phytol residue and the central Mg by chlorophyllase occurs at the initial reaction in the Chl breakdown pathway. Chlorophyllase genes, termed *CLHs*, were reported to be involved in Chl breakdown [26], but recent studies questioned the involvement of CLHs in Chl degradation in vivo during leaf senescence [23,27]. In 2009, functional analysis of pheophytinase (PPH) supports its role in porphyrin-phytol hydrolysis involved in senescence-related chlorophyll breakdown in vivo [28]. Several reports have revealed that the resulting pheophorbide (pheide) a is converted into a primary fluorescent chlorophyll catabolite (pFCC) in the next-step reactions, and two enzymes are involved in the reactions, including pheide a oxygenase (PAO) and red chl catabolite reductase (RCCR) [29,30,31,32]. *PAO* encodes a Rieske-type iron-sulfur oxygenase that is bound to chloroplast envelop, also named as *ACCELERATED CELL DEATH* (*ACD1*) [30]. PAO functions as a key enzyme that catalyzing the cleavage of the porphyrin ring in Chl breakdown pathway, and the red chlorophyll catabolite resulted from reactions can be further catalyzed into pFCC by RCCR. Next, a primary active transport system is responsible for exporting the resulting primary fluorescent catabolite pFCCs from the plastid and importing into the vacuole [33,34]. Based on the genetic analysis of the stay-green mutants, *NON-YELLOW COLORING1* (*NYC1*) and *NYC1-LIKE* (*NOL*) were cloned and found to selectively retain photosystem II (PSII) light-harvesting complex subunits [35,36]. The first half of chlorophyll *b* can be catalyzed into chlorophyll *a* by chlorophyll *b* reductase that is composed of two subunits NYC1 and NOL [36]. In addition, leaves of the loss-of-function mutant *pao* showed a stay-green phenotype under dark-induction conditions and a light-dependent lesion mimic phenotype was also observed because of the increase in levels of phototoxic pheide *a* [31,37,38]. Interestingly, the *Bf993* mutant of *Festuca pratensis* is classified into the third type of stay-green mutants in which the stay-green gene named as *senescence-induced degradation* (*SID*) is defective [39,40,41]. *SGR* (*STAY-GREEN*), designated as the orthologous gene of *SID*, has been cloned and characterized in a lot of plant species, including *Arabidopsis* [42], rice [43,44,45], pea [45,46], bell pepper [47,48], and tomato [47]. Based on the accumulated evidence, the direct interacting relationship has been characterized to exist between SGR and a subset of the protein components of the light harvesting chlorophyll *a/b*-protein complex II (LHCPII), suggesting that SGR is likely to play a role in making pigment-protein complexes unstable as a prerequisite for the enzymes in chlorophyll breakdown pathway to reach their substrate when leaves are in senescing processes [22,44,49].

A visible sign of leaf senescence and fruit ripening, loss of green color is resulted from massive Chl breakdown into nonphototoxic breakdown products in combination with carotenoid retention or anthocyanin accumulation [22,23,50]. Under normal growth conditions, chlorophyll maintains at a steady level due to the balance of its biosynthesis and degradation without a visible change in chlorophyll content [23,25]. In contrast, chlorophylls undergo turnover or breakdown when the partial or complete dismantling of the photosynthetic machinery occurs in response to environmental stresses, including heat stress. Heat stress symptoms in plants are typically characterized by leaf senescence or chlorosis due to a decline in chlorophyll content [2,51,52]. Heat-induced leaf chlorosis has been observed in a variety of plant species, including *Arabidopsis* [4,5,53], Soybean (*Glycine max* L. Merr.) [54], sorghum (*Sorghum bicolor*) [55], wheat (*Triticum aestivum*) [56,57] and creeping bentgrass (*Agrostis stolonifera*) [58]. However, the underlying mechanisms by which leaf senescence is regulated during heat stress remain elusive. Further studies are needed for answering the question of whether heat-induced chlorophyll loss is caused by heat-induced inhibition of chlorophyll synthesis and/or heat-enhanced chlorophyll degradation in plant leaves.

In *Arabidopsis*, chlorophyll *a* (Chl *a*) content was gradually reduced and chlorophyllase (Chlase) activity substantially increased during the high temperature treatment [53]. Upon heat treatment, no significant difference was detected in the enzyme activity of a key chlorophyll-synthesizing enzyme, porphobilinogen deaminase across all the lines of bentgrass (*Agrostis* spp.). However, the activities of chlorophyll-degrading enzymes, including chlorophyllase and chlorophyll-degrading peroxidase, increased significantly after heat stress whereas pheophytinase activity was unchanged [52]. Interestingly, lower activities of chlorophyll-degrading enzymes were detected in heat-tolerant transgenic lines in which the expression of isopentenyl transferase (*ipt*) gene is driven by a senescence-activated promoter (*SAG12*) or heat shock promoter (*HSP18.2*) in modulation of cytokinin biosynthesis when compared with the WT under heat stress [52]. The authors suggested that the enhanced degradation of chlorophyll under heat stress could result in a severe loss of the chlorophyll content in heat-challenged bentgrass. Studies on genetic variations contrasting in heat tolerance in the strength of leaf senescence under heat stress in hybrids of colonial (*Agrostis capillaris*) x creeping bentgrass (*Agrostis stolonifera*) supported that heat-induced loss of chlorophyll in leaves was caused by the rapid breakdown of chlorophyll, as manifested by the high-level activation of genes encoding chlorophyllase and pheophytinase, and the activity of pheophytinase (PPH) [59]. Recently, the map-based cloning of a semidominant, heat-sensitive, missense allele (*cld1-1*) led to identify a putative hydrolase, named as CHLOROPHYLL DEPHYTYLASE1 (CLD1) that is capable of dephytylating chlorophyll [60]. Their findings suggest that CLD1 is conserved in oxygenic photosynthetic organisms and plays a key role as the long-sought enzyme in making the phytol chain removed from chlorophyll in its degradation process at the steady-state level in chloroplasts.

Unlike studies on hormonal regulation of leaf senescence, less attention has been paid to effects of hormones on heat-induced chlorophyll loss. Exogenous application of a synthetic form of cytokinin, zeatin riboside (ZR) helped maintain higher leaf chlorophyll content creeping bentgrass exposed to heat stress by slowing down the action of protease and by induction or upregulation of heat-shock proteins [61]. The ethylene-inhibiting compound 1-methylcyclopropene (1-MCP) treatment can delay leaf senescence in cotton plants under high temperature by reducing lipid peroxidation, membrane leakage, soluble sugar content, and increasing chlorophyll content [62]. It was reported that the novel ethylene antagonist, 3-cyclopropyl-1-enyl-propanoic acid sodium salt (CPAS), increases grain yield in wheat by delaying leaf senescence under extreme weather conditions [63].

Plant chlorophyll retention-staygreen is considered a valuable trait under heat stress. Accumulating data indicate that understanding the physiological and molecular mechanisms of “STAY-GREEN” trait or delayed leaf senescence is required for regulating photosynthetic capability and may be also a key to break the plateau of productivity associated with adaptation to high temperature [64]. Staygreen traits are associated with heat tolerance in bread wheat and quantitative trait loci (QTL) for staygreen and related traits were identified across the genome co-located with agronomic and physiological traits associated to plant performance under heat stress, confirming that the staygreen phenotype is a useful trait for productivity enhancement in hot-irrigated environments [65]. In soybean, high temperatures and drought stress can lead to chlorophyll retention in mature seeds, termed as “green seed problem”, which is usually related to lower oil and bad seed quality, thus inducing a yield loss of soybean seeds. A “mild” stay-green phenotype was observed in a susceptible soybean cultivar when subjected to combined abiotic stresses of heat and drought stress and also the transcript levels of the *STAY-GREEN 1* and *STAY-GREEN 2* (*D1*, *D2*), *PHEOPHORBIDASE 2* (*PPH2*) and *NON-YELLOW COLORING 1* (*NYC1*) genes were downregulated in soybean seeds, indicating that the high-level transcriptional activation of these genes mentioned above in fully mature seeds is critical for a tolerant cultivar to cope with stresses and conduct a rapid and complete turnover of chlorophyll [66].

It is well-established that the major responses of crop plants to heat stress are classified into several aspects including the enhancement of leaf senescence, reduction of photosynthesis, deactivation of photosynthetic enzymes, and generation of oxidative damages to the chloroplasts. With respect to crop yield, heat stress also reduces grain number and size by affecting grain setting, assimilate translocation and duration and growth rate of grains [57]. In wheat, delayed senescence, or stay-green, contributes to a long grain-filling period and stable yield under heat stress [67]. Waxy maize (*Zea mays* L. sinensis Kulesh) is frequently exposed to high temperatures during grain filling in southern China. The heat-sensitive waxy maize variety exhibited a significant increase in the translocation amount and rate of assimilates pre-pollination and the accelerated leaf senescence phenotype under heat stress [68]. Short heat waves during grain filling can reduce grain size and consequently yield in wheat (*Triticum aestivum* L.). The four susceptible varieties of wheat showed greater heat-triggered reductions in final grain weight, grain filling duration and chlorophyll contents in flag leaves under heat stress, suggesting that grain size effects of heat may be driven by premature senescence [69,70].

## 3. Generation and Homeostasis of ROS in Chloroplasts under Heat Stress

As a significant source of reactive oxygen species (ROS) in plant cells, the chloroplast produces a variety of ROS such as hydrogen peroxide (H_2_O_2_), superoxide, hydroxyl radicals (^•^OH), and ^1^O_2_ during photosynthesis [71]. The transfer of excitation energy in the PSII antenna complex and the electron transport in the PSII reaction center can be inhibited by a variety of abiotic stresses, resulting in the formation of ROS in algae and higher plants [72]. Given that ROS generation is an unavoidable consequence of aerobic metabolism, plants have evolved a large array of ROS-scavenging mechanisms [71]. ROS such as ^1^O_2_ is formed by the excitation energy transfer, whereas superoxide anion radical (O_2_^•−^), H_2_O_2_ and ^•^OH are formed by the electron transport [73]. In chloroplasts, ROS are mainly generated in the reaction centers of PSI and PSII in the chloroplast thylakoid membranes [71]. Photoreduction of oxygen to superoxide occurs in PSI and in PSII, oxygen of the ground (triplet) state of oxygen (^3^O_2_) is excited to the excited singlet state of oxygen (^1^O_2_) by the P680 reaction center chlorophyll [71]. Under extreme conditions, these ROS synthesis rates can increase, leading to an oxidative stress in both organelle and whole-cell functions [72,74]. It is well established that the chloroplast is extremely sensitive to high temperature stress during photosynthesis [1,2,9,10]. Accumulated data indicate that oxidative bursts of superoxide and/or hydrogen peroxide can be induced rapidly in response to a variety of environmental stresses, including also heat stress in plants [75,76,77]. It was reported that ROS, produced in PSI, PSII as well as in the Calvin-Benson cycle, can cause irreversible oxidative damage to cells when plants were subjected to heat stress [71,72,78]. Under high temperature conditions, large amounts of ROS were generated in tobacco cells for initiating signaling events involved in PCD, which is consistent with the role of applying the antioxidants ascorbate or superoxide dismutase (SOD) to the cultures in supporting the survival of cells [79]. In the leaves of tobacco (*Nicotiana tabacum*) defective in ndhC-ndhK-ndhJ (Delta ndhCKJ), hydrogen peroxide was rapidly produced in response to heat treatment, implying a role of the NAD(P)H dehydrogenase-dependent pathway in repressing generation of reactive oxygen species in chloroplasts [80]. In exposure to moderate heat treatment conditions, the oxidative damages of the reaction center-binding D1 protein of photosystem II increased and showed a tight positive relationship with the accumulated levels of ^1^O_2_ and ^•^OH in spinach PSII membranes, implying that inhibition of a water-oxidizing manganese complex led to a rapid production of ROS through lipid peroxidation under heat stress [81]. In *Arabidopsis*, large amounts of chlorophyllide *a* caused a surge of phototoxic singlet oxygen in the chlorophyll synthase mutant (*chlg-1*) under heat stress, suggesting that chlorophyll synthase acts in maintenance of ROS homeostasis in response to heat stress [82].

It is well known that ROS burst can trigger the oxidative damage to pigments, proteins, and lipids in the thylakoid membrane [71,73,83]. ROS are primarily agents of damage, but this view is questioned by data proving their beneficial role and particularly their signaling function. The chloroplast harbors ROS-producing centers (triplet chlorophyll, ETC in PSI and PSII) and a diversified ROS-scavenging network (antioxidants, SOD, APX-glutathione cycle, and a thioredoxin system) to keep the equilibrium between ROS production and scavenging [71]. The non-enzymatic and enzymatic ROS scavenging systems are engaged in preventing harmful effects of ROS on the thylakoid membrane components to keep ROS level in chloroplasts under control [71,73,74,83,84,85,86,87]. The efficient enzymatic scavenging systems are composed of several key enzymes, including superoxide dismutase (SOD), catalase (CAT), ascorbate peroxidase (APX), glutathione reductase (GR), monodehydroascorbate reductase (MDHAR), dehydroascorbate reductase (DHAR), glutathione peroxidase (GPX) and glutathione-*S*-transferase (GST) and non-enzymatic systems contain antioxidants such as ascorbic acid (ASH), glutathione (GSH), phenolic compounds, alkaloids, non-protein amino acids and alpha-tocopherols [21,71,73,83,87,88,89,90]. These antioxidant defense systems work in concert to maintain homeostasis of ROS, protecting plant cells from oxidative damage by scavenging of ROS.

In addition to triggering ROS burst, heat stress also affects the scavenging systems in plants. Heat induced degradation of chloroplast Cu/Zn superoxide dismutase as shown by reduced protein levels and isozyme-specific SOD activity [91]. Loss of Cu/Zn SOD and induction of catalase activity would explain the altered balance between hydrogen peroxide and superoxide under stress. The authors proposed that degradation of PSII could thus be caused by the loss of components of chloroplast antioxidant defense systems and subsequent decreased function of PSII [91]. In a recent study aiming at identifying heat protective mechanisms promoted by CO_2_ in coffee crop, the results likely favored that the maintenance of reactive oxygen species (ROS) at controlled levels contributed to mitigate of PSII photoinhibition under the high temperature stress [92]. Exogenous application of spermidine protected rice seedlings from heat-induced damage as marked by lower levels of malondialdehyde (MDA), H_2_O_2_, and proline content coupled with increased levels of Ascorbate (AsA), GSH, AsA and GSH redox status [93]. The authors conclude that heat exposure provoked an oxidative burden while enhancement of the antioxidative and glyoxalase systems by spermidine rendered rice seedlings more tolerant to heat stress. Under the later high temperature stress, heat priming contributed to a better redox homeostasis, as exemplified by the higher activities of superoxide dismutase (SOD) in chloroplasts and glutathione reductase (GR), and of peroxidase (POD) in mitochondria, which led to the lower superoxide radical production rate and malondialdehyde concentration in both chloroplasts and mitochondria [94]. These results suggested that heat priming effectively improved thermo-tolerance of wheat seedlings subjected to a later high temperature stress, which could be largely ascribed to the enhanced anti-oxidation at the subcellular level. In Kentucky bluegrass (*Poa pratensis*), higher activities of superoxide dismutase (SOD), catalase (CAT), peroxidase (POD), ascorbate peroxidase (APX), and glutathione reductase were detected in plants of heat-tolerant “Midnight” after long-term heat stress (21 and 28 d) in comparison with plants of heat-sensitive “Brilliant” [95]. Meanwhile, transcript levels of chloroplastic Cu/Zn SOD, Fe SOD, CAT, POD and cytosolic (cyt) APX were significantly higher in “Midnight” than in “Brilliant” under long-term heat stress. These results supported the hypothesis that enzymatic ROS scavenging systems could play predominant roles in antioxidant protection against oxidative damages from long-term heat stress. In tomato, studies on the role of a tomato (*Lycopersicon esculentum*) chloroplast-targeted DnaJ protein (LeCDJ1) showed that the sense transgenic tomato plants were more tolerant to heat stress due to the higher activities of ascorbate peroxidase (APX) and superoxide dismutase (SOD) [96]. The high temperature stress in southern China is one of the major factors leading to loss of the yield and quality of wucai (*Brassica campestris* L.). Comparative investigations on two cultivars of wucai (heat-sensitive and heat-tolerant) showed that greater severity of damage to the photosynthetic apparatus and membrane system was observed in heat-sensitive cultivar probably due to a high-level accumulation of ROS and malondialdehyde (MDA) [97]. In line with the studies mentioned above, plants of heat-tolerant Cucurbit species exhibited comparatively little oxidative damage, with the lowest hydrogen peroxide (H_2_O_2_), superoxide (O^2−^) and malondialdehyde (MDA) compared with the thermolabile and moderately heat-tolerant interspecific inbred line [98]. The enzyme activities of superoxide dismutase (SOD), ascorbate peroxidase (APX), catalase (CAT) and peroxidase (POD) were found to be increased with heat stress in tolerant genotypes and the significant inductions of FeSOD, MnSOD, APX2, CAT1 and CAT3 isoforms in tolerant genotypes suggested their participation in heat tolerance [98].

**Figure 2:**
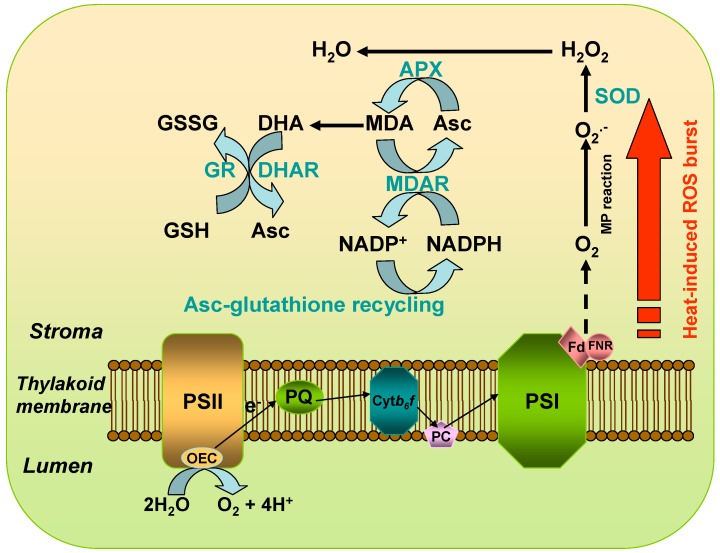
A representative scheme of reactive oxygen species (ROS) generation and scavenging in chloroplasts under heat stress. High temperature stress triggers oxidative bursts of superoxide and/or hydrogen peroxide in plants. The transfer of excitation energy in the photosystem II (PSII) antenna complex and the electron transport in the PSII reaction center can be inhibited by heat stress. It has been established that ROS are generated both on the electron acceptor and the electron donor side of PSII under heat stress during which electron transport from the manganese complex to plastoquinone (PQ) is limited. The leakage of electrons to molecular oxygen on the electron acceptor side of PSII forms O_2_^•−^, inducing initiation of a cascade reaction leading to the formation of H_2_O_2_. A diversified ROS-scavenging network functions in concert in chloroplasts, mainly including antioxidants and APX-glutathione cycle, to keep the equilibrium between ROS production and scavenging. The efficient enzymatic scavenging systems are composed of several key enzymes, including superoxide dismutase (SOD), ascorbate peroxidase (APX), glutathione reductase (GR), monodehydroascorbate reductase (MDHAR), dehydroascorbate reductase (DHAR), glutathione peroxidase (GPX) and glutathione-*S*-transferase (GST) and non-enzymatic systems contain antioxidants such as ascorbic acid (Asc) and glutathione (GSH).

Generally, the chloroplast possesses a variety of constitutively expressed antioxidant defense mechanisms to scavenge the various types of ROS generated during heat stress in preventing and minimizing oxidative damage to biological macromolecules (Figure 2). Therefore, the capacity of antioxidant defense is critical for heat stress adaptation and its strength is correlated with acquisition of thermotolerance with respect to buffering the effect of heat stress on the metabolic system [3,74,99,100]. On the other hand, ROS are produced in chloroplasts can function as plastid signals to inform the nucleus to activate the expression of genes encoding antioxidant enzyme and to adjust the stress-responsive machinery for more efficient adaptation to environmental stresses [6,47,72,74,100,101].

## 4. Turnover of PSII Core Subunits and PSII Protection under Heat Stress

The deleterious effects of high temperatures on proteins in chloroplasts include protein denaturation and aggregation [18,102,103]. ROS generation is one of the earliest responses of plant cells in response to heat stress and chloroplasts are the main targets of ROS-linked damage [71,102]. Given that chloroplasts are a major site of protein degradation, turnover of the damaged proteins is critical for plants to adapt to heat stress through the process of acclimation. As a multisubunit thylakoid membrane pigment-protein complex, PSII is vulnerable to light and heat damages that inhibit light-driven oxidation of water and reduction of plastoquinone [2,71]. PSII produces ROS, responsible for the frequent damage and turnover of this megacomplex that occur under physiological and stress conditions [71]. Accumulating data suggest that although more than 40 proteins are known to associate with PSII, the damage mainly targeted to one of its core proteins, the D1 protein under light and heat stress conditions [18,102,104,105]. Importantly, D1 protein has been demonstrated as a very susceptible target of ^1^O_2_; on the other hand, it appears to function as a major scavenger of ^1^O_2_ due to its close localization to the site of ^1^O_2_ formation in the reaction center of PSII [106]. In addition to the D1 protein, β-carotene, plastoquinol, and a-tocopherol have also shown to play a role in scavenging ^1^O_2_ and protect PSII against photo-oxidative damage [107,108]. Under heat stress conditions, two processes have been characterized in the thylakoids: dephosphorylation of the D1 protein in the stroma thylakoids, and aggregation of the phosphorylated D1 protein in the grana [102]. Heat stress also induced the release of the extrinsic Photosystem II Subunit O (PsbO), P and Q proteins from Photosystem II, which affected D1 degradation and aggregation significantly [102]. In spinach thylakoids, cleavage of the D1 protein was detected in response to moderate heat stress (40 °C, 30 min), producing an N-terminal 23-kDa fragment, a C-terminal 9-kDa fragment, and aggregation of the D1 protein [102,103]. ROS are known to specifically modify PSII proteins. Using high-resolution tandem mass spectrometry, oxidative modifications were identified on 36 amino acid residues on the lumenal side of PSII, in the core PSII proteins D1, D2, and CP43 of the cyanobacterium *Synechocystis* sp. PCC 6803, providing the compelling evidence to date of physiologically relevant oxidized residues in PSII [109]. Taken together, the oxidative damage of the D1 protein is caused by reactive oxygen species, mostly singlet oxygen, and also by endogenous cationic radicals generated by the photochemical reactions of photosystem II [71,73]. Under heat stress, the damage to the D1 protein by moderate heat stress is due to reactive oxygen species produced by lipid peroxidation near photosystem II [73,110]. Moreover, the damage of the D1 protein has been shown to be directly proportional to light intensity [111,112] or strength of heat stress [102]. D1 protein is well characterized as a protein of high turnover rate due to its rapid degradation when oxidized after its interaction with 1O2 and its replacement by newly synthesized D1 polypeptides [105]. It was reported that the D1 protein was shown to have the half-life of 2.4 h, the fourth fastest turn-over rate of barley (*Hordeum vulgare*) proteins when plants were growing under normal growth light intensity (500 µmol · m^−2^ · s^−2^) [113]. Meanwhile, higher degradation rates of the D2, CP43 and PsbH subunits were also detected when compared with the other PSII subunits [113,114,115]. The maintenance of PSII activity is critical, but also a challenge for oxygenic photosynthetic organisms to survive under normal growth or stress conditions.

Thus, replacing the photo- or heat-damaged D1 with a newly synthesized copy is essential for maintaining PSII activity [105,116]. In chloroplasts, the damaged D1 is degraded by a concerted action of particular filamentation temperature sensitive H (FtsH) protease and Deg (for degradation of periplasmic proteins) isoforms during its rapid and specific turnover and replaced with a de novo synthesized one in a system which is termed the PSII repair cycle [105]. It has been a subject of extensive studies about the basic concept for replacement of the damaged D1 protein by a newly-synthetized copy in the PSII repair cycle [105,116,117,118,119]. Repair of damaged D1 protein in PSII includes five steps: (1) migration of damaged PSII core complex to the stroma thylakoid, (2) partial PSII disassembly of the PSII core monomer, (3) access of protease degrading damaged D1, (4) concomitant D1 synthesis, and (5) reassembly of PSII into grana thylakoid [105,116,120,121]. Deg /HtrA (for high temperature requirement A) proteases, a family of serine-type ATP-independent proteases, have been shown in higher plants to be involved in the degradation of the Photosystem II reaction center protein D1 [105,116,122]. In *Arabidopsis*, five DEGs (Deg1, Deg2, Deg5, Deg7, and Deg8) have shown to be peripherally attached to the thylakoid membrane of chloroplasts: Deg1, Deg5, and Deg8 are localized on the lumenal side, and Deg2 and Deg7 are localized on the stromal side [123,124,125]. DEG5 and DEG8 may have synergistic function in degradation of D1 protein in the repair cycle of PSII under heat stress based on functional analysis of *deg5*, *deg8* and the double mutant *deg5 deg8* of *Arabidopsis thaliana* [126]. Under photoinhibitory conditions, cooperative degradation of D1 by Deg and FtsH has been demonstrated in vivo, in which Deg cleavage assists FtsH processive degradation [127]. In Arabidopsis, FtsH11-encoded protease have shown to play a direct role in thermotolerance, a function previously reported for bacterial and yeast FtsH proteases [128]. It should be noted that photosynthetic capability and PSII quantum yield are greatly reduced in the leaves of FtsH11 mutants when exposed to the moderately high temperature whereas under high light conditions, FtsH11 mutants and wild-type plants showed no significant difference in photosynthesis capacity [128]. In general, several possible mechanisms have been proposed for activation of these proteases, which depend on oligomerization of the monomer subunits [129]. In line with the hypothesis that hexamers of the FtsH proteases are probably localized near the Photosystem II complexes at the grana, degradation of the D1 protein could take place in the grana rather than in the stroma thylakoids to circumvent long-distance migration of both the Photosystem II complexes containing the photodamaged D1 protein and the proteases [129]. Under high light conditions, the lumen-exposed loops of the D1 protein at specific sites were cleaved by all of three lumenal serine proteases, Deg1, Deg5 and Deg8, during PSII repair cycle [130,131,132,133]. It was reported that Deg5 and Deg8 interact to form an active protease complex under high light [133]. Interestingly, Deg1 is activated when the Deg1 monomers are transformed into a proteolytically active hexamer at acidic pH upon protonation of a histidine amino acid residue [134]. In addition to functioning as a protease, Deg1 also plays a novel role as a chaperone/assembly factor of PSII [135]. In addition, Deg1 has been shown to be responsible for the proteolytic activity against the PsbO protein in vitro [136]. During the PSII repair cycle, only the damaged reaction center protein D1 and occasionally also the D2, CP43 and PsbH subunits are replaced while the other protein components of the complex are recycled, indicating that many aspects of PSII repair cycle and de novo biogenesis are partially overlapping [113,114,115,137]. Originally, the vulnerability of PSII to high light or heat stress was taken as an inherent fault of the photosynthetic machinery. However, recent studies strongly support that the constant, yet highly regulated, photodamage and repair cycle of PSII are of a strong physiological basis. Collectively, the photodamage of PSII is likely to act as a PSI protection mechanism instead of being considered solely as an undesired consequence of the highly oxidizing chemistry of the water splitting PSII [105].

The synthesis of heat shock proteins (HSPs) is characterized as a major response of all organisms responding to heat stress. The HSPs act as chaperones by assisting in protein folding and preventing irreversible protein aggregation [138,139]. A chloroplast-localized sHSP, HSP21, has been identified in diverse higher plant species, including both dicots and monocots [139,140] and its precursor polypeptide is ~5 kD larger than the mature protein [141,142]. HSP21 is thought to protect photosynthetic electron transport, specifically that of Photosystem II, during heat stress [143,144,145,146,147] and oxidative stress [145,148]. In addition, HSP21 has been demonstrated to play a dual role in protecting PSII from oxidative stress and promoting color changes during fruit maturation whereas no protective effects for the transgene were detected on PSII thermotolerance [149]. Importantly, studies using the transgenic tomato plants overexpressing HSP21 have shown that this protein associates with proteins of Photosystem II and does not reactivate heat-denatured Photosystem II, but instead protects this complex from damage during heat stress [150]. Interestingly, around two thirds of chloroplast HSP21 proteins are translocated into the thylakoid membranes in response to heat treatment in plants, suggesting that the association with membranes should be considered to fully understand the role of sHsps in physiological adaptation processes under stress conditions [151]. Despite extensive studies on HSP21, the specific roles of HSP21 in protecting PSII from heat stress remain elusive. Recently, HSP21 has been demonstrated to protect PSII from heat stress-induced damages by directly binding to D1 and D2 proteins, the core subunits of PSII. Importantly, heat-responsive transcriptional activation of *HSP21* is regulated by the chloroplast retrograde signaling pathway in which GUN5 acts as a determinant upstream signaling component in *Arabidopsis* [5]. Based on these findings, an auto-adaptation loop working module has emerged in which the GUN5-dependent plastid signal(s) is triggered in response to heat stress and in turn communicated into the nucleus to activate the heat-responsive expression of *HSP21* for optimizing particular demands of chloroplasts in making photosynthetic complexes stable during adaptation to heat stress in plants [5,6].

## 5. Effects of Heat Stress on Metabolic Flux through the Calvin-Benson-Bassham Cycle

In the Calvin-Benson-Bassham Cycle, Ribulose-1,5-bisphosphate carboxylase/oxygenase (Rubisco) plays a critical role in catalyzing the carboxylation of the 5-carbon sugar ribulose-1,5-bisphosphate (RuBP) when atmospheric CO_2_ during is fixed during photosynthesis. Rubisco activase (RCA) regulates the activity of Rubisco by facilitating the dissociation of inhibitory sugar phosphates from the active site of Rubisco in an ATP-dependent manner [152]. Extensive evidence supports the conclusion that reduction of plant photosynthesis arises primarily from thermal inactivation of Rubisco activity due to the inhibition of RCA under moderately elevated temperatures [8,152,153,154,155]. In addition to Rubisco activation, electron transport activity, ATP synthesis, and RuBP regeneration are also inhibited by moderately heat stress [156,157,158]. As the temperature increases further above the thermal optimum, the physical integrity of electron transport components of the photosynthetic apparatus can be severely damaged, leading to the increased limitation in photosynthesis [15]. It has been the subject of extensive investigations of elucidating the biochemical basis for the decrease in Rubisco activation state under heat stress [8,15,153,154,159]. Initially, studies on thermal stability of purified RCA showed that heat treatment only slightly inhibited the activities of this enzyme [160,161] and later experiments confirmed that heat stress caused thermal denaturation of activase in wheat and cotton leaves [162]. In line with the thermal denaturation of RCA under heat stress, Feller et al. (1998) suggested that RCA exhibited the exceptional thermal lability in vivo and the thermal stabilities of activases were different in plants from contrasting thermal environments [15,159]. Thus, loss of activase activity during heat stress is caused by an exceptional sensitivity of the protein to thermal denaturation and is responsible, in part, for deactivation of Rubisco [163]. It has been assumed that the stability of RCA could be influenced by heat-induced changes either in redox state [11,164,165] or the concentrations of ions, nucleotides, or other chloroplast constituents in plants [154]. On the other hand, the thermotolerance of Rubisco activase has been proposed to be responsible for restricting the distribution of certain plant species [154] as demonstrated by the response of Rubisco activase activity to temperature for cotton, a warm-season species, and *Camelina sativa*, a cool-season species. With respect to the effects of high growth temperature on the relative contribution of diffusive and biochemical limitations to photosynthesis, our knowledge is limited although there is abundant evidence that photosynthesis can acclimate to temperature [166,167]. Accumulating data suggest that the biochemical mechanisms about the decrease in Rubisco activation can be attributed to: (1) more rapid de-activation of Rubisco caused by a faster rate of dead-end product formation; and (2) slower re-activation of Rubisco by activase [168]. In a word, the resulting consequence is that RCA becomes less effective in keeping Rubisco catalytically competent as temperature increases.

Inhibition of net photosynthesis by heat stress has been attributed to an inability of Rubisco activase to maintain Rubisco in an active form because of the low thermal stability of Rubisco's chaperone, activase. These results support a role for RCA in limiting photosynthesis at high temperature when the temperature exceeds the optimum range for plants. In cotton (*Gossypium hirsutum* L.), activase gene expression is influenced by post-transcriptional mechanisms that may contribute to acclimation of photosynthesis during extended periods of heat stress [169]. In wheat, northern blot analysis showed maximum accumulation of *TaRCA1* transcript in thermotolerant cv. during mealy-ripe stage, as compared to thermosusceptible ones [170]. To test the hypothesis that thermostable RCA can improve photosynthesis under elevated temperatures, gene shuffling technology was used to generate several *Arabidopsis thaliana* RCA1 (short isoform) variants exhibiting improved thermostability [171]. In line with the findings mentioned above, transgenic Arabidopsis lines expressing a thermostable chimeric activase showed higher rates of photosynthesis than the wild type after a short exposure to higher temperatures and they also recovered better, when they were returned to the normal temperature [172]. The results showed that photosynthesis and growth were improved under moderate heat stress in transgenic Arabidopsis expressing these thermotolerant RCA isoforms, providing evidence that manipulation of activase properties can improve C3 photosynthesis. In addition, the transcriptional level of wheat *RCA* (45–46 kDa) positively correlated with the yield of plants under heat-stress conditions in a very significant and linear manner [173]. At present, accumulating data indicate that RCA could affect plant productivity in relation to its endogenous levels under temperature stress conditions. Critically, RCA as the molecular chaperone plays a key role in constant engagement and remodeling of Rubisco to maintain metabolic flux through the Calvin-Benson-Bassham cycle as Rubisco is characterized as a dead-end inhibited complex in higher plants. In plants of the crassulacean acid metabolism (CAM), it has been assumed that possessing thermostable RCA is necessary for these plants to support the metabolic flux of Calvin-Benson-Bassham cycle when closure of stomata is a limitation factor during the day [174]. It is interesting that the CAM Rca isoforms (*Agave tequilana*) were found to be approximately 10 °C more thermostable when compared with the C3 isoforms of Rca isolated from rice (*Oryza sativa*) [174]. Interestingly, sequence analysis and immuno-blotting identified the beta-subunit of chaperonin-60 (cpn60 beta), the chloroplast GroEL homologue, as a protein that was bound to Rubisco activase from leaf extracts prepared from heat-stressed, but not control plants [175]. Rubisco requires RCA, an AAA+ ATPase that reactivates Rubisco by remodeling the conformation of inhibitor-bound sites. RCA is regulated by the ratio of ADP:ATP, with the precise response potentiated by redox regulation of the alpha-isoform [176]. Given that RCA uses the energy from ATP hydrolysis to restore catalytic competence to Rubisco, manipulation of RCA by redox regulation of the a-isoform might provide a strategy for enhancing photosynthetic performance in *Arabidopsis* [177]. In rice, heat stress significantly induced the expression of *RCA* large isoform (*RCAL*) as determined by both mRNA and protein levels and correlative analysis indicated that and RCA small isoform (RCAS) protein content was very tightly correlated to Rubisco initial activity and net photosynthetic rate under both heat stress and normal conditions [178]. In two *Populus* species adapted to contrasting thermal environments, the difference in the primary sequence of Rubisco activases between the species is more significant in the regions conferring ATPase activity and Rubisco recognition, suggesting that the genotypic distinctive characterizations in Rubisco activase are likely to underlie the specificities with respect to the heat-sensitive strength of Rubisco activase and photosynthesis under moderate high temperature conditions [179]. Recent studies on the effects of heat and drought on three major cereal crops, including rice, wheat, and maize, indicate that reductions in Rubisco activation might be not dependent on the amount of Rubisco and RCA, but could be resulted from the inhibition of RCA activity, as evidenced by the mutual reduction and positive relationship existed between the activation state of Rubisco and the rate of electron transport [153]. Critically, Rubisco activase acts as a key player in photosynthesis under heat stress conditions (non-stomatal limitation) [180]. When exposed to a moderate heat stress, Rca can be inhibited reversibly, but is irreversibly inhibited under a higher temperature and/or longer exposure due to heat stress-induced insolubilization and degradation of the Rca protein [180].

## 6. Conclusions and Perspectives

In many regions of the world, high temperature stress is one of the most important constraints to plant growth and productivity, especially for crop plants. The mechanism underlying the development of heat-tolerance for important agricultural crops as well as plant responses and adaptation to elevated temperatures needs to be better understood. Extensive studies have shown that metabolic regulation of adaptation processes during heat stress is not only an important developmental process, but also allows for flexibility of physiological responses to heat stress. In photosynthetic organisms, heat stress can affect photosynthesis through altered carbon assimilation metabolism in chloroplasts with remobilizing their starch reserve to release energy, sugars and derived metabolites in order to help mitigate the stress. This is thought to be an essential process for plant fitness with important implications for plant productivity under high temperature stress. One future challenges is to dissect the complex interaction networks between heat stress sensing, signal transduction and activations of key genes involved in metabolic reprogramming in coordination with developmental programmes. Accumulation and modification of metabolites in chloroplasts under heat stress may play a key role in the regulation of adaptation processes at cellular levels in plants, allowing plants to interact with their environment and to activate cellular heat stress responses at the optimal time in order to maintain photosynthesis. This kind of metabolic reprogramming is critical for plants to survive stress periods, and to prevent further damage to the whole plant.

The role of chloroplast in the metabolic regulation of heat stress responses has attracted increasing attention and extensive investigations from an organellar perspective have provided insights into better understanding the hypothesis stated that the heat stress-induced reprogramming, including decline in photosynthesis and alterations in photosynthetic metabolites which, in turn, could act as signal(s) or trigger the initial signal cascades to activate cellular heat stress responses. The present knowledge concerning the interplay between the chloroplast and nucleus in heat stress signal perception and activation of cellular heat stress responses is emerging, but more efforts are needed to reach a detailed overview. It can be predicted that uncovering the molecular mechanisms of heat sensing will pave the way to engineering plants capable of tolerating heat stress. It is well known that the ability of plants adapting to different climate regimes vary dramatically across and within species. Identification and functional analysis of the valuable heat-tolerant genetic resources will bring about a further significant improvement in manipulation of photosynthesis to increase crop yield based on a direct comparative analysis between the different manipulations with all the transgenic and wild-type plants grown and assessed in parallel under filed growth conditions. Thus, in-depth analyses of the interactions between the chloroplast and nucleus in heat stress responses are likely to be in focus during forthcoming years. On the other hand, Rubisco activase and enzymes functioning in the detoxification of reactive oxygen species are thought to be critical targets for breeding heat-tolerant crop plants with high yields under high temperature stress.

## Abbreviations

1-MCP	1-methylcyclopropene
^1^O_2_	singlet state of oxygen
^3^O_2_	triplet state of oxygen
ACD1	Accelerated cell death
APX	ascorbate peroxidase
AsA	Ascorbate
ASH	ascorbic acid
ATP	adenosine 5′-triphosphate
CAM	crassulacean acid metabolism
CAT	catalase
Chl	Chlorophyll
Chl *a*	Chlorophyll *a*
Chlase	chlorophyllase
CLD1	CHLOROPHYLL DEPHYTYLASE1
CLHs	Chlorophyllase genes
CPAS	3-cyclopropyl-1-enyl-propanoic acid sodium salt
cpn60 beta	beta-subunit of chaperonin-60
Deg/HtrA	high temperature requirement A
DHAR	dehydroascorbate reductase
FtsH	filamentation temperature sensitive H
Fv/Fm	variable fluorescence to maximum fluorescence
GPX	glutathione peroxidase
GR	glutathione reductase
GSH	glutathione
GST	glutathione-*S*-transferase
GUN5	genomes uncoupled 5
H_2_O_2_	hydrogen peroxide
HSPs	heat shock proteins
ipt	isopentenyl transferase
LeCDJ1	tomato (*Lycopersicon esculentum*) chloroplast-targeted DnaJ protein
LHCPII	light harvesting chlorophyll *a*/*b*-protein complex II
MDA	malondialdehyde
MDHAR	monodehydroascorbate reductase
NAD(P)H	nicotinamide adenine dinucleotide phosphate (NADP)
NOL	NYC1-LIKE
NYC1	NON-YELLOW COLORING1
O_2_^•−^	superoxide anion radical
OEC	oxygen evolving complex
OH	hydroxyl radicals
PAO	pheide a oxygenase
pFCC	primary fluorescent chlorophyll catabolite
pheide	pheophorbide
POD	peroxidase
PPH	pheophytinase
PPH2	PHEOPHORBIDASE 2
PsbO	PHOTOSYSTEM II SUBUNIT O
PSII	Photosystem II
QTL	quantitative trait loci
RCA	Rubisco activase
RCAL	RCA large isoform
RCAS	RCA small isoform
RCCR	Red chl catabolite reductase
ROS	Reactive oxygen species
Rubisco	Ribulose-1,5-bisphosphate carboxylase/oxygenase
RuBP	Ribulose-1,5-bisphosphate
SGR	STAY-GREEN
SID	senescence-induced degradation
SOD	superoxide dismutase
ZR	zeatin riboside
